# Health Tract, No. 22

**Published:** 1868-02

**Authors:** 


### REGULATING THE BOWELS.

It is best that the bowels should act every morning after breakfast; therefore, quietly remain in the house, and promptly attend to the first inclination. If the time passes, do not eat an atom until they do act; at least not until breakfast next day, and even then do not take any thing except a single cup of weak coffee or tea, and some cold bread and butter, or dry toast, or ship-biscuit.

Meanwhile, arrange to walk or work moderately, for an hour or two, each forenoon and afternoon, to the extent of keeping up a moisture on the skin, drinking as freely as desired as much cold water as will satisfy the thirst, taking special pains, as soon as the exercise is over, to go to a good fire or very warm room in winter, or, if in summer, to a place entirely sheltered from any draught of air, so as to cool off very slowly indeed, and thus avoid taking cold or feeling a "soreness" all over next day.

Remember, that without a regular daily healthful action of the bowels, it is impossible to maintain health, or to regain it if lost. The coarser the food, the more freely will the bowels act, such as corn (Indian) bread eaten hot, hominy, wheaten grits, bread made from coarse flour, or "shorts," Graham bread, boiled turnips, or stirabout, or grapes, or dried figs, or stewed tamarinds. A handful or two of boiled or raw chestnuts eaten during the day; a tablespoonful, more or less, thrice a day of white mustard-seed swallowed whole, in water or otherwise; eating freely of parched corn; taking, on rising, a tumblerful of cream which has been allowed to stand until it has thickened, whether sweet or sour, are means which are sometimes successful in keeping the bowels acting freely once a day, without the necessity of taking medicine. When one fails to keep up a good effect, try another; in the hope that when the bowels have got into a habit of regular action, it may be kept up by the judicious employment of such daily food as observation may show is best adapted to the object. The habitual use of pills, or drops, or any kind of medicine whatever, for the regulation of the bowels, is a sure means of ultimately undermining the health; in almost all cases laying the foundation for some of the most distressing of chronic maladies; hence, all the pains possible should be taken to keep them regulated by natural agencies, such as the coarse foods and exercises above named, or stewed prunes, or a glass of water on rising, into which has been stirred a teaspoonful of salt, or a heaping tablespoonful of corn meal. Reliance on injections is disastrous eventually.

If the bowels act oftener than twice a day, live for a short time on boiled rice, farina, starch, or boiled milk. In more aggravated cases, keep as quiet as possible on a bed, take nothing but rice, parched brown like coffee, then boiled and eaten in the usual way; meanwhile drink nothing whatever, but eat to your fullest desire bits of ice swallowed nearly whole, or swallow ice-cream before entirely melted in the mouth; if necessary, wear a bandage of thick woolen flannel, a foot or more broad, bound tightly round the abdomen; this is especially necessary if the patient has to be on the feet much. All locomotion should be avoided when the bowels are thin, watery, or weakening.

